# Overlooked histories in ageing research: Pioneering women at the foundation of our field

**DOI:** 10.1111/acel.14432

**Published:** 2024-11-28

**Authors:** Marina Ezcurra, Colin Selman, Jennifer Tullet, Nathan Woodling

**Affiliations:** ^1^ School of Biosciences University of Kent Canterbury UK; ^2^ School of Molecular Biosciences University of Glasgow Glasgow UK

**Keywords:** biogerontology, epigenetics, gender equality, geroscience, geriatrics, nutrition, oxidative damage, women in science

## Abstract

A list of this decade's most prominent names in ageing research would undoubtedly include many women who have led the field in recent years. While the field, and science in general, still have far to go in achieving gender parity in opportunities and recognition, we can celebrate the progress made to date. However, the longer ‘history of the field’ that many of us present in our classrooms, conference halls and writings often tends to be dominated by men. Although numerous men have made fundamental observations that have shaped our understanding of ageing from both a mechanistic and evolutionary perspective, the unfortunate reality is that women making similar contributions have not received equal recognition throughout much of our field's history. As a starting point for wider representation and further conversations in this area, we present here a short list of women—Marjory Warren, Lillian Jane Martin, Margaret Alexander Ohlson, Rebeca Gerschman and Marion J. Lamb—whose contributions were foundational to ageing research in the 20th century. Their work spanned theoretical, experimental and clinical insight into the biology of ageing—and yet their names are too seldom mentioned when introducing our field. We hope this list can be a starting point for a more inclusive recognition of the diverse scientists who helped pave the way for our field today.

## Graphical abstract

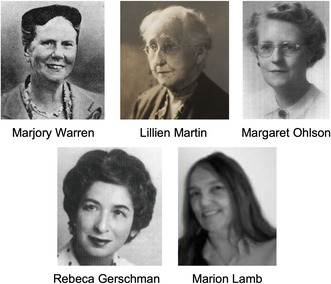

## 1 INTRODUCTION

**FIGURE 1:**
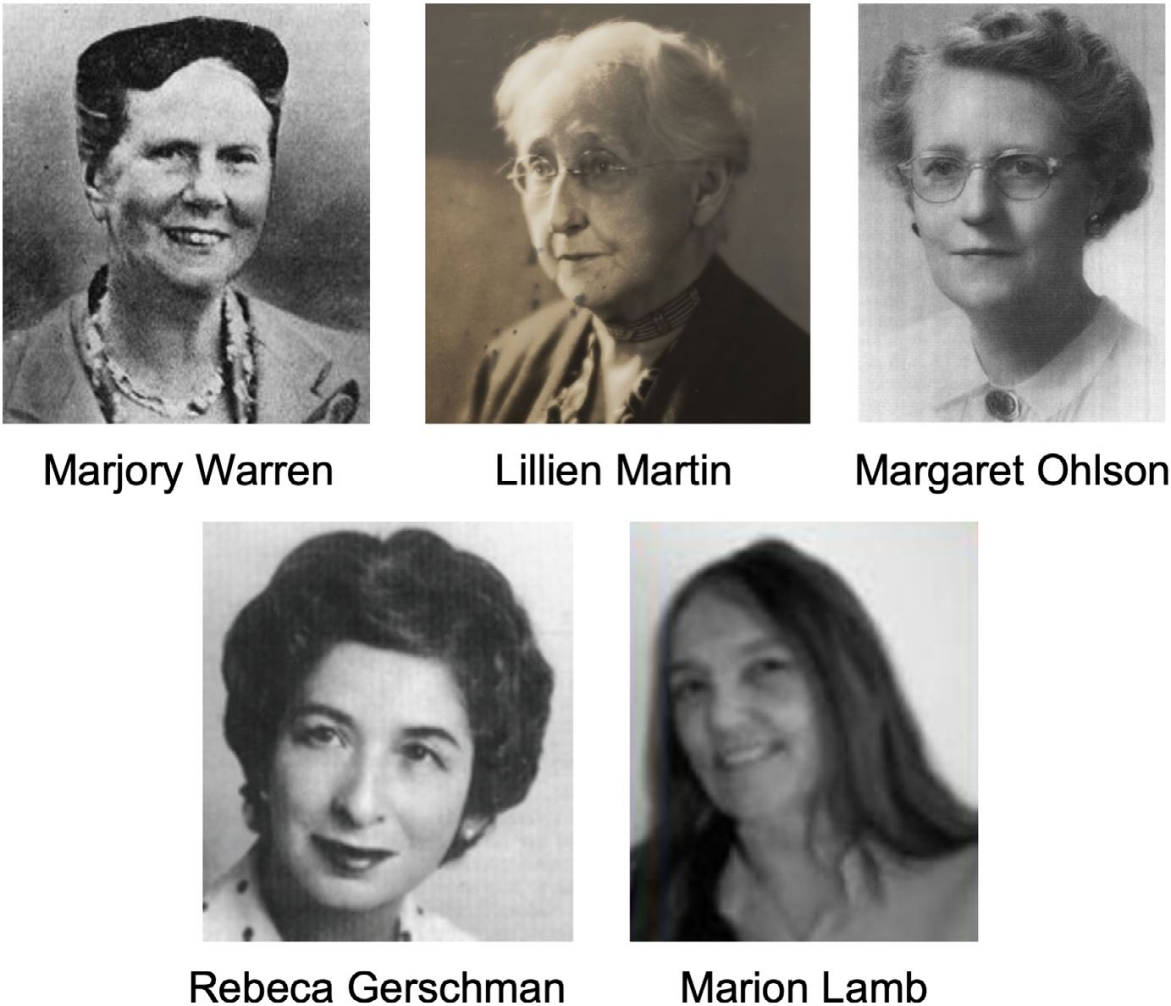
Pioneering women in ageing research. Photo credits: Marjory Warren, *Photo*: *Archives of the British Geriatrics Society*; Lillien Martin, courtesy of the Department of Special Collections, Stanford University Libraries, SC1071, Stanford Historical Photograph Collection, Box 17; Margaret Ohlson, image reproduced with permission from The Journal of the Academy of Nutrition and Dietetics (Wenberg & Lechowich, 1997); Rebeca Gerschman, photo free of access from https://sebbm.es/mujer‐y‐ciencia/rebeca‐gerschman/; Marion Lamb, from https://alchetron.com/Marion‐J‐Lamb.

Research on the biology of ageing has undergone a revolution in the last few decades. Evolutionarily conserved pathways and processes central to ageing have been identified, and there is increasing evidence that ageing trajectories can be modified. Among scientists and wider society, hope is growing that biogerontology research can be translated into interventions that improve lifelong health and well‐being. This rapid progress has been driven by the discoveries and insights of many scientists worldwide. A significant proportion of these are women, but particularly in the early part of the 20th century, many did not receive the recognition they deserved. Here we describe the innovative and groundbreaking work of a small group of pioneering women who were ahead of their time: advancing geriatric care, trailblazing personalised nutrition and laying the mechanistic foundations of biogerontology. We share their overlooked histories to celebrate their contributions and enhance representation in biogerontology research (Figure 1).

## 2 PIONEERING GERIATRIC MEDICINE AND GERONTOLOGY

Populations are ageing globally, and the accompanying age‐related multimorbidity is placing a rapidly growing burden on healthcare systems. The need to address the medical complexities of older patients means geriatric medicine is becoming more important than ever; however, recognition of this urgent and unmet need is far from new. Marjory Warren (1897–1960) laid the foundation of geriatrics and envisioned transforming healthcare of older patients with treatments targeted at age‐specific requirements (Denham, 2011). She was ahead of her time in distinguishing between ageing and disease and understood that ageing was associated with multimorbidity and impaired resilience.

In the late 1930s, Warren set out to transform a ‘care’ system that often referred to older‐age patients as ‘inmates’, typically held in dim, cramped and poorly organised wards (St John & Hogan, 2014). She pioneered geriatrics by carefully categorizing patients according to their needs, improving ward conditions and highlighting the importance of space for activities and recreation. Her landmark papers highlighted that older adults needed specialised treatment distinct from that of younger patients, with considerations for age‐specific needs such as diet and medical equipment (Warren, 1943). She further emphasised the need for specialist geriatric care by collating statistics on the then‐current and projected rise in older populations in both the UK and USA (Warren, 1946). Her proposals emphasised that medicine's success in combating disease not only led to an increase in life expectancy but also created an urgent demand to meet the specific medical needs of older patients. While Warren's work (primarily based at the West Middlesex Municipal Hospital) attracted some national attention from the UK Ministry of Health, the societal impediments of being a woman and the lack of an advanced medical degree meant that she was not fully acknowledged in her own time (St John & Hogan, 2014). Nevertheless, her foundational work in geriatrics continues to influence modern healthcare systems that still confront many of the same challenges Warren championed.

Lillien Jane Martin (1851–1943) was a pioneering and transformative American clinical and experimental psychologist who also had a profound interest in the well‐being of older adults. She recognised the importance of healthy living in combating the effects of old age. After becoming the first woman to head an academic department at Stanford University in 1900, she established the San Francisco Old Age Center, the first dedicated old‐age counselling centre in the USA. By the time of her death in 1943, over 3000 clients had benefitted from its gerontology‐specific counselling methods, which helped encourage the development of new interests and skills into old age (Terman, 1949). Martin wrote several books arguing that, although advances in medicine had extended life expectancy, older people were still constrained by conventions and stereotypes that limited their opportunities to lead fulfilling life. She not only developed psychiatric approaches to help older adults live gratifying lives (Martin, 1944) but also followed her own teachings, learning to drive at 78 years of age and travelling the Andes and Amazon at age 88. Despite her significant contributions to psychology and her inspirational life experiences, Martin remains relatively obscure, making her ‘one of psychology's best kept secrets’ (Stevens & Gardner, 1982).

## 3 PIONEERING PERSONALISED NUTRITION FOR HEALTHY AGEING

There is a growing recognition that ageing alters nutritional requirements and that lifespan and healthspan can be modified by dietary interventions. The concept that individuals have different ageing trajectories which could be altered through targeted nutritional interventions (personalised nutrition) is an active research area with a rich, yet sometimes overlooked, history. Margaret Alexander Ohlson (1901–1996) was a pioneer in nutrition, metabolism and ageing in humans. She understood early on that nutritional requirements vary between individuals and change with age. Ohlson earned a PhD in Clinical Nutrition from the University of Iowa and later headed the Food and Nutrition Department as Professor of Internal Medicine at Michigan State College. Her approach was highly collaborative, meticulous and holistic, featuring long‐term physical and biochemical investigations underpinned by detailed information on participants' home environment, dietary intake and medical history.

Ohlson's early research explored the complexities of weight‐loss diets and nutritional deficiencies in women (Brown & Ohlson, 1946), and she later conducted seminal work on the dietary requirements of ageing women. Her studies were intensive, typically involving daily visits to the participants' homes to build ‘friendships’ and maximise participant involvement (Ohlson, 1948). Her efforts demonstrated that nutritional deficiency negatively impacts the capacity for productive work without limiting lifespan. Among her other significant contributions, she highlighted the importance of calcium supplementation during ageing, emphasised the challenges of extrapolating data from rodents to humans, and advocated for specific diets to manage different disease states in both children and adults. In her final publication, she presented a longitudinal study involving 158 women followed over a 37‐year period, meticulously documenting changes in calorie and nutrient intake across the life course (Ohlson & Harper, 1976). She received the Borden Award for Research in Human Nutrition and the Marjorie Hulsizer Copher Award from the American Dietetic Association. After retiring to Seattle, the city where she spent her childhood, she remained active by undertaking early computer science research at the University of Washington and volunteering for a homeless charity.

## 4 LAYING THE FOUNDATIONS OF MOLECULAR BIOGERONTOLOGY

In parallel with early work on geriatric healthcare and diet, ageing research began to develop mechanistic theories to explain the ageing process. Rebeca Gerschman (1903–1986) played a central role in this new mechanistic understanding of ageing: her work revolutionised thinking around reactive oxygen species toxicity, antioxidant defences, and their relevance to age‐related molecular damage. Her studies on respiratory gases advanced the biological understanding of antioxidants years before the field fully developed, leading her to hypothesise that antioxidants are critical for ageing (Boveris, 1996).

Born in Argentina to Russian Jewish immigrants, Gerschman studied in Buenos Aires and pursued a PhD in physiology under the supervision of Nobel laureate Bernard Houssay, during which she established a colorimetric method to measure potassium levels in plasma (Gerschman‐Marenzi method). Following World War II, Gerschman worked with Wallace Fenn at the University of Rochester. In post‐war United States there was a growing interest in understanding the effects of oxygen and radiation on health, prompted by advancements in aviation and military medicine. Gerschman discovered similarities between the effects of high concentrations of oxygen and ionising radiation, leading her to propose that both were caused by a common mechanism, an increase in reactive oxygen species. She further proposed the existence of endogenous mechanisms (antioxidants) that protect against both oxygen and ionising radiation. This work, published in *Science* (Gerschman et al., 1954), initially generated much debate and scepticism. Notably, it predates Denham Harman's much more famous and frequently cited publication, ‘Aging: A theory based on free radical and radiation chemistry’ (Harman, 1956), which does not cite Gerschman's work. According to Google Scholar, Harman's publication has been cited more than 12,000 times as of September 2024—while Gerschman's paper has received just over 1400 citations. While some of this disparity may be due to Harman's specific focus on ageing as opposed to Gerschman's broader focus, it is worth considering what other factors might have led to her work being less well‐known.

Gerschman continued studying the toxicity of oxygen, using a comparative biology approach utilizing rodents, bacteria, fungi, protozoans and plants—all now recognised as essential tools in biogerontology research. Her early findings proved remarkably accurate regarding the role of reactive oxygen species in many pathological states and helped demonstrate the conservation of protective mechanisms that organisms have evolved to protect against oxidative damage (Cornejo, 2015). The discovery of superoxide dismutase in 1969 confirmed Gerschman's theory (McCord & Fridovich, 1969). By that time Gerschman had returned to Buenos Aires as Professor of Physiology, where she continued her research on respiratory gases while innovating teaching practises, such as showing films of physiological experiments (Boveris, 1996). Gerschman was known for her strong and magnetic personality, and active academic and social life. The parties she hosted at her home were famous in Buenos Aires academic circles. She retired in 1980 and died in 1986.

In addition to oxidative damage‐linked theories of ageing, biogerontology began to consider genetic and epigenetic mechanisms that contribute to ageing, drawing on major advances in genetics during the mid‐20th century. Marion J. Lamb (1939–2021) played a key role as a pioneer in epigenetics: her work challenged traditional Darwinian perspectives and explored how environmental factors can affect gene expression across generations. Lamb completed her PhD in 1965 with the theoretical geneticist and evolutionary biologist John Maynard Smith at University College London (UCL). Following this, she worked at UCL, Harwell and Birkbeck, where she held the post of senior lecturer (equivalent to associate professor in the US academic system) until her retirement (Jablonka, 2022).

Lamb's work on epigenetic inheritance influenced contemporary views on genetic and epigenetic contributions to evolution and ageing. Her book ‘Evolution in Four Dimensions’ co‐authored with Eva Jablonka (Jablonka & Lamb, 2005), proposed new theories integrating epigenetic mechanisms with classical genetics, offering a nuanced view of heredity and evolution. Lamb studied how genetics and environmental conditions—such as heat, radiation and pollution—affect metabolic activity and mutagenesis in the fruit fly *Drosophila*. She explored how epigenetic changes accumulate over an organism's lifespan and contribute to age‐related functional decline. Her work suggests that ageing could be partially understood through epigenetic modifications disrupting normal cellular functions and leading to age‐related phenotypic manifestations. Her framework posited that ageing results from the interplay between genetic predispositions and epigenetic changes influenced by environment and lifestyle. Lamb thus challenged the view that ageing is solely due to genetic mutations and cellular wear and tear, emphasizing that a dynamic genome modulated by epigenetic factors in response to external stimuli can influence lifespan. Today, epigenetic alterations are considered a hallmark of ageing (López‐Otín et al., 2023), and Lamb's early insights were central to the development of this field.

Lamb had a sharp intellect and commitment to scientific integrity. She was described as ‘ferocious and incredibly kind’, disliking what she called ‘little academic games'’ (Jablonka, 2022). Her modesty and dislike for ‘undeserved credit’ meant that she turned down many offered (and warranted) co‐authorships during her academic career. She was dedicated to clarity in scientific communication, as exemplified in her beautifully written book *The Biology of Ageing* (Lamb, 1977). Much of its content holds true today, and the impeccable clarity with which it explains difficult concepts has made it a valuable resource for courses on ageing biology at institutions worldwide.

## 5 CONCLUDING THOUGHTS

Here, we have presented a few short profiles to highlight the significant contributions of these five women to the field of ageing research. There are, of course, many other women, as well as individuals from other minoritised groups, whose crucial contributions to ageing research have been overlooked and whose stories deserve to be heard. In our teaching and in the stories we tell about our field, we all have a responsibility to acknowledge and celebrate the diversity of scientists who have helped pave the way for the advancements we see today.

## AUTHOR CONTRIBUTIONS

Conceptualisation: M.E., C.S., J.T. and N.W. Investigation: M.E., C.S., J.T. and N.W. Writing (original draft): M.E., C.S., J.T. and N.W. Writing (review and editing): M.E., C.S., J.T. and N.W.

## CONFLICT OF INTEREST STATEMENT

None declared.

## Data Availability

Data sharing is not applicable to this article as no new data were created or analyzed in this study.
